# *rac*-4-(4-Chloro­phen­yl)-2-methyl­amino-3-nitro-5,6,7,8-tetra­hydro-4*H*-chromen-5-one

**DOI:** 10.1107/S1600536813014530

**Published:** 2013-06-08

**Authors:** P. Narayanan, Jayabal Kamalraja, Paramasivam T. Perumal, K. Sethusankar

**Affiliations:** a Department of Physics, RKM Vivekananda College (Autonomous), Chennai 600 004, India; b Organic Chemistry Division, Central Leather Research Institute, Adyar, Chennai 600 020, India

## Abstract

The title compound, C_16_H_15_ClN_2_O_4_, contains a chiral centre and crystallizes as a racemate. The methyl­ene group β-positioned to the carbonyl group is partially (21%) disordered. It flips to the opposite sides of the corresponding six-membered carbocycle by −0.304 (3) and 0.197 (11) Å, producing alternative envelope conformations. The planes of the pyran and chloro­phenyl rings form a dihedral angle of 86.25 (9)°. The mol­ecular structure is characterized by an intra­molecular N—H⋯O inter­action, which generates an *S*(6) ring motif. The corresponding amino N atom deviates from the plane of the pyran ring by 0.1634 (19) Å. In the crystal, mol­ecules are linked *via* C—H⋯O hydrogen bonds, forming *C*(8) chains running parallel to the *b*-axis direction. The crystal structure also features C—H⋯π inter­actions.

## Related literature

For the uses and biological importance of chromene, see: Ercole *et al.* (2009 ▶); Geen *et al.* (1996 ▶) Khan *et al.* (2010 ▶); Raj *et al.* (2010 ▶). For related structures, see: Sun *et al.*, (2012 ▶). For graph-set notation, see: Bernstein *et al.* (1995 ▶). 
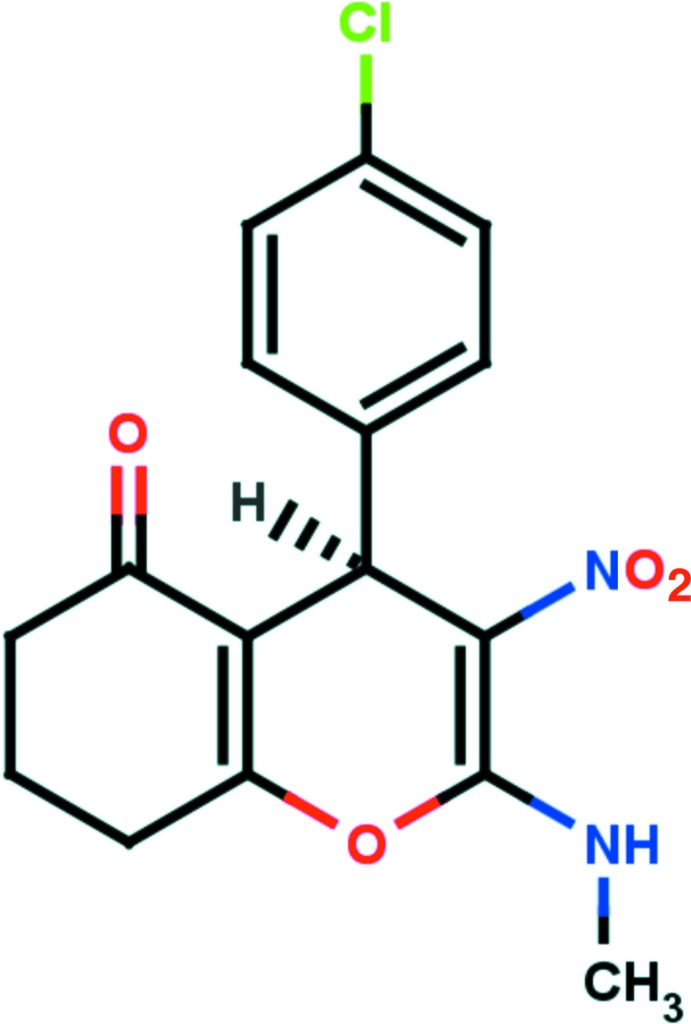


## Experimental

### 

#### Crystal data

C_16_H_15_ClN_2_O_4_
*M* *_r_* = 334.75
Monoclinic, 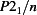

*a* = 8.0285 (4) Å
*b* = 10.8460 (5) Å
*c* = 18.2337 (9) Å
β = 94.067 (2)°
*V* = 1583.74 (13) Å^3^
*Z* = 4
Mo *K*α radiation
μ = 0.26 mm^−1^
*T* = 296 K
0.35 × 0.30 × 0.30 mm

#### Data collection

Bruker SMART APEXII CCD diffractometer
Absorption correction: multi-scan (*SADABS*; Bruker, 2008 ▶) *T*_min_ = 0.912, *T*_max_ = 0.924
11848 measured reflections
2786 independent reflections
2208 reflections with *I* > 2σ(*I*)
*R*_int_ = 0.029

#### Refinement

*R*[*F* ^2^ > 2σ(*F* ^2^)] = 0.042
*wR*(*F* ^2^) = 0.119
*S* = 1.09
2786 reflections
218 parameters
4 restraints
H atoms treated by a mixture of independent and constrained refinement
Δρ_max_ = 0.32 e Å^−3^
Δρ_min_ = −0.28 e Å^−3^

### 

Data collection: *APEX2* (Bruker, 2008 ▶); cell refinement: *APEX2*; data reduction: *SAINT* (Bruker, 2008 ▶); program(s) used to solve structure: *SHELXS97* (Sheldrick, 2008 ▶); program(s) used to refine structure: *SHELXL97* (Sheldrick, 2008 ▶); molecular graphics: *ORTEP-3* (Farrugia, 2012 ▶); software used to prepare material for publication: *SHELXL97* and *PLATON* (Spek, 2009 ▶).

## Supplementary Material

Crystal structure: contains datablock(s) global, I. DOI: 10.1107/S1600536813014530/ld2104sup1.cif

Structure factors: contains datablock(s) I. DOI: 10.1107/S1600536813014530/ld2104Isup2.hkl

Supplementary material file. DOI: 10.1107/S1600536813014530/ld2104Isup3.cml

Additional supplementary materials:  crystallographic information; 3D view; checkCIF report

## Figures and Tables

**Table 1:** Hydrogen-bond geometry (Å, °) *Cg*1 is the centroid of the pyran ring C7/C8/C13/O1/C14/C15.

*D*—H⋯*A*	*D*—H	H⋯*A*	*D*⋯*A*	*D*—H⋯*A*
N2—H2*A*⋯O3	0.90 (2)	1.86 (2)	2.599 (2)	137 (2)
C2—H2⋯O4^i^	0.93	2.53	3.420 (3)	160
C10—H10*A*⋯*Cg*1^ii^	0.97	2.75	3.515 (2)	136
C16—H16*B*⋯*Cg*1^iii^	0.96	2.76	3.577 (3)	144

Symmetry codes: (i) 
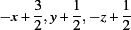
; (ii) 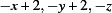; (iii) 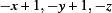.

**Fig. 1.**
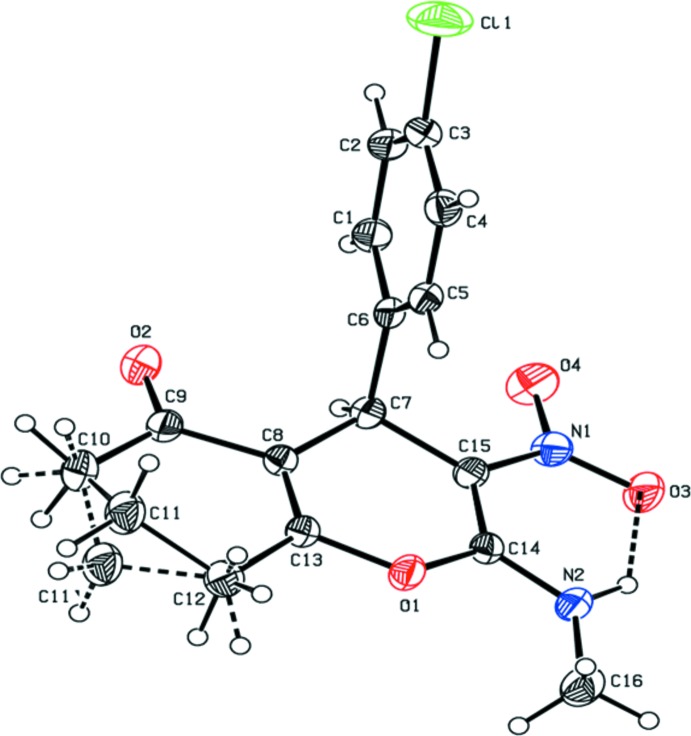
The molecular structure of the title compound, with the atom-numbering scheme. The intramolecular hydrogen bond is shown. The displacement ellipsoids are drawn at 30% probability level. H atoms are shown as spheres of arbitary radius.

**Fig. 2.**
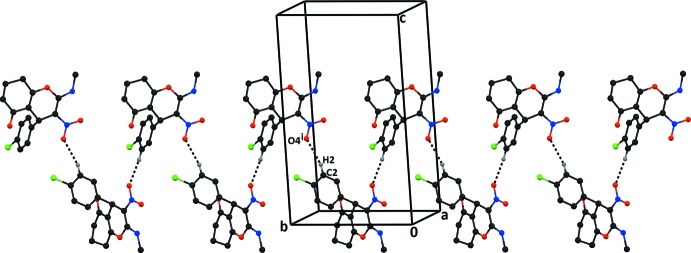
The crystal packing of the title compound, viewed along c axis, showing C2—H2···O4i hydrogen bonds producing C(8) chains parallel to b axis. H atoms not involved in the hydrogen bonding have been excluded for clarity. The symmetry code: (i) 3/2 - x, 1/2 + y, 1/2 - z.

## supplementary crystallographic information

### Comment

Chromene derivatives are very important heterocyclic compounds that have a variety of industrial, biological and chemical synthesis applications (Geen *et al.*, 1996; Ercole *et al.*, 2009). They exhibit a number of pharmacological activities such as anti-HIV, anti-inflammatory, anti-bacterial, anti-allergic, anti-cancer *etc.* (Khan *et al.*, 2010, Raj *et al.*, 2010). Against this backround, X-ray analysis of the title compound has been carried out to study its structural aspects.

X-ray analysis confirms the molecular structure and atom connectivity. The molecular structure is stabilized by intramolecular N2—H2A···O3 interaction, which generates *S*(6) ring motif as illustrated in Fig. 1. The methelene group carbon atom C11 of the chromene moiety is disordered over two positions, with an occupancy factor of 0.787 (5):0.213 (5). The pyrane ring (C7/C8/C13/C14/C15/O1) is almost orthogonal to the chlorophenyl ring (C1–C6), with a dihedral angle of 86.25 (9)° between their mean planes.

The pyrane ring is almost coplanar with the least-square planes of the nitro and methylene groups, making dihedral angles of 5.19 (14) and 5.01 (16)°, with them, respectively.

The six-membered carbocyclic rings (C8/C9/C10/C11/C12/C13) and (C8/C9/C10/C11'/C12/C13) of the chromene moiety adopt envelope conformations on the atoms C11 and C11', with puckering paramaters: Q_2_ = 0.366 (3) Å, Q_3_ = 0.229 (3) Å and φ_2_ = 178.1 (2)°, and Q_2_ = 0.211 (7) Å, Q_3_ = -0.185 (5) Å and φ_2_ = 3.1 (2)°, respectively. Also, the atoms C11 and C11' deviate from their respective mean planes of the rest of the ring atoms by -0.304 (3) and 0.197 (11) Å, respectively. The amine group nitrogen atom N2 deviates by 0.1634 (19) Å from the mean plane of the pyran ring. The chlorine atom Cl1 deviates from the phenyl ring (C1–C6) by 0.0571 (9) Å. The title compound exihibits structural similarities with an already reported related structure (Sun *et al.*, 2012).

In the crystal, the molecules are linked *via* intermolecular C2—H2···O4^i^ hydrogen-bond interaction, which generates *C*(8) chains running parallel to *b* axis (Bernstein *et al.*,1995). The crystal structure is further stabilized by C10—H10A···*Cg*1^ii^ and C16—H16B···*Cg*1^iii^ intermolecular interactions, where *Cg*1 is the center of gravity of the pyran ring (C7/C8/C13/O1/C14/C15). The symmetry codes: (i) 3/2 - *x*, 1/2 + *y*, 1/2 - *z* (ii) 2 - *x*, 2 - *y*, -*z* (iii) 1 - *x*, 1 - *y*, -*z*. The packing view of the title compound is shown in Fig. 2.

### Experimental

A solution of 4-chlorobenzaldehyde (0.14 g, 1.0 mmol), cyclic 1,3-dicarbonyl compound (1.0 mmol), NMSM (0.15 g, 1.0 mmol) and piperidine (0.2 equivalents) in EtOH (2 ml) was stirred for 3.5 h. After the reaction was complete as indicated by TLC, the product was filtered and washed with EtOH (2 ml) to remove the excess base and other impurities. Single crystals suitable for X-ray diffraction were prepared by slow evaporation of a solution of the title compound in ethanol at room temperature.

### Refinement

Positions of the H atoms were localized from the difference electron-density maps and their distances were geometrically constrained. The H atoms of the amine group were constrained to distances of N—H = 0.901 (10) Å with *U*_iso_(H) = 1.2*U*_eq_(N). The H atoms bound to the C atoms were treated as riding atoms, with C—H = 0.93 Å and *U*_iso_(H) = 1.2*U*_eq_(C) for aromatic, C—H = 0.97 Å and *U*_iso_(H) = 1.2*U*_eq_(C) for methelene, C—H = 0.98 Å and *U*_iso_(H) = 1.2*U*_eq_(C) for methiene, and C—H = 0.96 Å and *U*_iso_(H) = 1.5 *U*_eq_(C) for methyl groups. The rotation angles for methyl groups were optimized by least squares. The bond distances of the disordered components were restrained using standard similarity restraint SADI (*SHELXL97*; Sheldrick, 2008) with an s.u. of 0.01 Å. The atomic displacement parameters of the major and minor components were made similar using the constraint EADP.

### Crystal data

**Table:**

C_16_H_15_ClN_2_O_4_	*F*(000) = 696
*M**_r_* = 334.75	*D*_x_ = 1.404 Mg m^−^^3^
Monoclinic, *P*2_1_/*n*	Mo *K*α radiation, λ = 0.71073 Å
Hall symbol: -P 2yn	Cell parameters from 2208 reflections
*a* = 8.0285 (4) Å	θ = 2.2–25.0°
*b* = 10.8460 (5) Å	µ = 0.26 mm^−^^1^
*c* = 18.2337 (9) Å	*T* = 296 K
β = 94.067 (2)°	Block, colourless
*V* = 1583.74 (13) Å^3^	0.35 × 0.30 × 0.30 mm
*Z* = 4	

### Data collection

**Table:**

Bruker SMART APEXII CCD diffractometer	2786 independent reflections
Radiation source: fine-focus sealed tube	2208 reflections with *I* > 2σ(*I*)
Graphite monochromator	*R*_int_ = 0.029
ω and φ scans	θ_max_ = 25.0°, θ_min_ = 2.2°
Absorption correction: multi-scan (*SADABS*; Bruker, 2008)	*h* = −9→8
*T*_min_ = 0.912, *T*_max_ = 0.924	*k* = −12→12
11848 measured reflections	*l* = −15→21

### Refinement

**Table:**

Refinement on *F*^2^	Primary atom site location: structure-invariant direct methods
Least-squares matrix: full	Secondary atom site location: difference Fourier map
*R*[*F*^2^ > 2σ(*F*^2^)] = 0.042	Hydrogen site location: inferred from neighbouring sites
*wR*(*F*^2^) = 0.119	H atoms treated by a mixture of independent and constrained refinement
*S* = 1.09	*w* = 1/[σ^2^(*F*_o_^2^) + (0.0482*P*)^2^ + 0.9511*P*] where *P* = (*F*_o_^2^ + 2*F*_c_^2^)/3
2786 reflections	(Δ/σ)_max_ = 0.002
218 parameters	Δρ_max_ = 0.32 e Å^−^^3^
4 restraints	Δρ_min_ = −0.28 e Å^−^^3^

### Special details

**Table:**

Geometry. All e.s.d.'s (except the e.s.d. in the dihedral angle between two l.s. planes) are estimated using the full covariance matrix. The cell e.s.d.'s are taken into account individually in the estimation of e.s.d.'s in distances, angles and torsion angles; correlations between e.s.d.'s in cell parameters are only used when they are defined by crystal symmetry. An approximate (isotropic) treatment of cell e.s.d.'s is used for estimating e.s.d.'s involving l.s. planes.
Refinement. Refinement of *F*^2^ against ALL reflections. The weighted *R*-factor *wR* and goodness of fit *S* are based on *F*^2^, conventional *R*-factors *R* are based on *F*, with *F* set to zero for negative *F*^2^. The threshold expression of *F*^2^ > σ(*F*^2^) is used only for calculating *R*-factors(gt) *etc*. and is not relevant to the choice of reflections for refinement. *R*-factors based on *F*^2^ are statistically about twice as large as those based on *F*, and *R*-factors based on ALL data will be even larger.

### Fractional atomic coordinates and isotropic or equivalent isotropic displacement parameters (Å^2^)

**Table:**

	*x*	*y*	*z*	*U*_iso_*/*U*_eq_	Occ. (<1)
C1	0.8013 (3)	0.7871 (2)	0.15451 (11)	0.0393 (5)	
H1	0.8934	0.7510	0.1798	0.047*	
C2	0.7070 (3)	0.8723 (2)	0.19018 (12)	0.0440 (5)	
H2	0.7358	0.8944	0.2387	0.053*	
C3	0.5699 (3)	0.9234 (2)	0.15224 (13)	0.0443 (5)	
C4	0.5244 (3)	0.8923 (2)	0.08060 (13)	0.0426 (5)	
H4	0.4302	0.9271	0.0561	0.051*	
C5	0.6215 (2)	0.80814 (19)	0.04556 (11)	0.0359 (5)	
H5	0.5927	0.7870	−0.0031	0.043*	
C6	0.7614 (2)	0.75474 (18)	0.08203 (11)	0.0321 (4)	
C7	0.8702 (2)	0.66430 (18)	0.04219 (11)	0.0333 (5)	
H7	0.9648	0.6402	0.0761	0.040*	
C8	0.9376 (2)	0.72565 (18)	−0.02368 (11)	0.0340 (5)	
C9	1.0653 (3)	0.8231 (2)	−0.01071 (13)	0.0408 (5)	
C10	1.1262 (3)	0.8872 (2)	−0.07623 (15)	0.0568 (7)	
H10A	1.1514	0.9722	−0.0631	0.068*	0.787 (5)
H10B	1.2293	0.8485	−0.0888	0.068*	0.787 (5)
H10C	1.0732	0.9676	−0.0794	0.068*	0.213 (5)
H10D	1.2450	0.9012	−0.0664	0.068*	0.213 (5)
C11	1.0072 (4)	0.8859 (3)	−0.14166 (18)	0.0539 (9)	0.787 (5)
H11A	1.0619	0.9181	−0.1834	0.065*	0.787 (5)
H11B	0.9140	0.9397	−0.1331	0.065*	0.787 (5)
C11'	1.1015 (14)	0.8288 (11)	−0.1501 (5)	0.0539 (9)	0.213 (5)
H11C	1.1949	0.7744	−0.1572	0.065*	0.213 (5)
H11D	1.1009	0.8925	−0.1874	0.065*	0.213 (5)
C12	0.9406 (3)	0.7556 (2)	−0.16027 (13)	0.0488 (6)	
H12A	0.8457	0.7608	−0.1962	0.059*	0.787 (5)
H12B	1.0268	0.7070	−0.1813	0.059*	0.787 (5)
H12C	0.9528	0.6925	−0.1972	0.059*	0.213 (5)
H12D	0.8519	0.8105	−0.1787	0.059*	0.213 (5)
C13	0.8893 (3)	0.69552 (19)	−0.09213 (11)	0.0360 (5)	
C14	0.7355 (2)	0.52214 (18)	−0.05564 (11)	0.0344 (5)	
C15	0.7772 (2)	0.54932 (18)	0.01722 (11)	0.0329 (5)	
C16	0.6240 (4)	0.3970 (3)	−0.15987 (14)	0.0699 (9)	
H16A	0.5475	0.4562	−0.1823	0.105*	
H16B	0.5771	0.3158	−0.1655	0.105*	
H16C	0.7275	0.4004	−0.1831	0.105*	
N1	0.7340 (2)	0.46946 (16)	0.07175 (10)	0.0408 (4)	
N2	0.6539 (2)	0.42483 (17)	−0.08263 (10)	0.0442 (5)	
O1	0.77966 (19)	0.59923 (14)	−0.10937 (8)	0.0436 (4)	
O2	1.1201 (2)	0.84650 (17)	0.05139 (10)	0.0574 (5)	
O3	0.6480 (2)	0.37405 (14)	0.05646 (9)	0.0529 (4)	
O4	0.7809 (3)	0.49375 (16)	0.13643 (9)	0.0593 (5)	
Cl1	0.45353 (11)	1.03329 (9)	0.19602 (5)	0.0864 (3)	
H2A	0.621 (3)	0.377 (2)	−0.0458 (10)	0.058 (8)*	

### Atomic displacement parameters (Å^2^)

**Table:**

	*U*^11^	*U*^22^	*U*^33^	*U*^12^	*U*^13^	*U*^23^
C1	0.0421 (12)	0.0421 (12)	0.0334 (11)	−0.0032 (10)	0.0014 (9)	0.0005 (9)
C2	0.0523 (13)	0.0468 (13)	0.0341 (12)	−0.0107 (11)	0.0099 (10)	−0.0078 (10)
C3	0.0439 (12)	0.0403 (12)	0.0510 (14)	−0.0058 (10)	0.0186 (10)	−0.0095 (11)
C4	0.0342 (11)	0.0407 (12)	0.0533 (14)	−0.0023 (9)	0.0048 (10)	0.0005 (10)
C5	0.0362 (11)	0.0359 (11)	0.0354 (11)	−0.0074 (9)	0.0023 (8)	−0.0038 (9)
C6	0.0334 (10)	0.0292 (10)	0.0342 (11)	−0.0072 (8)	0.0056 (8)	−0.0008 (8)
C7	0.0329 (10)	0.0325 (11)	0.0342 (11)	−0.0016 (8)	−0.0002 (8)	0.0004 (9)
C8	0.0325 (10)	0.0301 (11)	0.0401 (12)	−0.0018 (8)	0.0065 (8)	−0.0025 (9)
C9	0.0336 (11)	0.0371 (12)	0.0520 (14)	−0.0031 (9)	0.0066 (10)	−0.0091 (10)
C10	0.0599 (15)	0.0415 (14)	0.0707 (17)	−0.0169 (12)	0.0173 (13)	−0.0015 (12)
C11	0.062 (2)	0.0432 (19)	0.0571 (19)	−0.0125 (14)	0.0095 (16)	0.0095 (15)
C11'	0.062 (2)	0.0432 (19)	0.0571 (19)	−0.0125 (14)	0.0095 (16)	0.0095 (15)
C12	0.0615 (15)	0.0446 (13)	0.0417 (13)	−0.0119 (11)	0.0136 (11)	0.0021 (10)
C13	0.0379 (11)	0.0303 (11)	0.0405 (12)	−0.0044 (9)	0.0084 (9)	−0.0011 (9)
C14	0.0345 (10)	0.0297 (10)	0.0399 (12)	−0.0027 (8)	0.0081 (8)	−0.0001 (9)
C15	0.0360 (10)	0.0271 (10)	0.0358 (11)	−0.0005 (8)	0.0044 (8)	0.0014 (8)
C16	0.087 (2)	0.076 (2)	0.0483 (16)	−0.0372 (16)	0.0119 (14)	−0.0187 (14)
N1	0.0514 (11)	0.0311 (10)	0.0404 (11)	0.0013 (8)	0.0063 (8)	0.0047 (8)
N2	0.0542 (11)	0.0378 (11)	0.0416 (11)	−0.0144 (9)	0.0102 (9)	−0.0070 (9)
O1	0.0555 (9)	0.0415 (9)	0.0341 (8)	−0.0178 (7)	0.0051 (7)	−0.0012 (7)
O2	0.0482 (10)	0.0634 (11)	0.0606 (12)	−0.0179 (8)	0.0035 (8)	−0.0148 (9)
O3	0.0695 (11)	0.0331 (9)	0.0572 (11)	−0.0129 (8)	0.0109 (8)	0.0050 (7)
O4	0.0923 (14)	0.0494 (10)	0.0353 (10)	−0.0065 (9)	−0.0008 (9)	0.0095 (8)
Cl1	0.0797 (5)	0.0901 (6)	0.0916 (6)	0.0240 (4)	0.0224 (4)	−0.0350 (5)

### Geometric parameters (Å, º)

**Table:**

C1—C6	1.383 (3)	C10—H10D	0.9700
C1—C2	1.386 (3)	C11—C12	1.540 (4)
C1—H1	0.9300	C11—H11A	0.9700
C2—C3	1.375 (3)	C11—H11B	0.9700
C2—H2	0.9300	C11'—C12	1.516 (8)
C3—C4	1.373 (3)	C11'—H11C	0.9700
C3—Cl1	1.742 (2)	C11'—H11D	0.9700
C4—C5	1.386 (3)	C12—C13	1.487 (3)
C4—H4	0.9300	C12—H12A	0.9700
C5—C6	1.390 (3)	C12—H12B	0.9700
C5—H5	0.9300	C12—H12C	0.9700
C6—C7	1.531 (3)	C12—H12D	0.9700
C7—C8	1.507 (3)	C13—O1	1.387 (2)
C7—C15	1.507 (3)	C14—N2	1.319 (3)
C7—H7	0.9800	C14—O1	1.354 (2)
C8—C13	1.321 (3)	C14—C15	1.379 (3)
C8—C9	1.480 (3)	C15—N1	1.381 (3)
C9—O2	1.212 (3)	C16—N2	1.444 (3)
C9—C10	1.494 (3)	C16—H16A	0.9600
C10—C11	1.475 (4)	C16—H16B	0.9600
C10—C11'	1.489 (8)	C16—H16C	0.9600
C10—H10A	0.9700	N1—O4	1.241 (2)
C10—H10B	0.9700	N1—O3	1.264 (2)
C10—H10C	0.9700	N2—H2A	0.901 (10)
			
C6—C1—C2	121.4 (2)	C12—C11—H11A	109.1
C6—C1—H1	119.3	C10—C11—H11B	109.1
C2—C1—H1	119.3	C12—C11—H11B	109.1
C3—C2—C1	118.5 (2)	H11A—C11—H11B	107.8
C3—C2—H2	120.7	C10—C11'—C12	113.0 (6)
C1—C2—H2	120.7	C10—C11'—H11C	109.0
C4—C3—C2	122.0 (2)	C12—C11'—H11C	109.0
C4—C3—Cl1	119.25 (19)	C10—C11'—H11D	109.0
C2—C3—Cl1	118.77 (18)	C12—C11'—H11D	109.0
C3—C4—C5	118.7 (2)	H11C—C11'—H11D	107.8
C3—C4—H4	120.7	C13—C12—C11'	114.3 (4)
C5—C4—H4	120.7	C13—C12—C11	109.3 (2)
C4—C5—C6	121.05 (19)	C13—C12—H12A	109.8
C4—C5—H5	119.5	C11'—C12—H12A	132.3
C6—C5—H5	119.5	C11—C12—H12A	109.8
C1—C6—C5	118.42 (19)	C13—C12—H12B	109.8
C1—C6—C7	120.98 (18)	C11'—C12—H12B	72.9
C5—C6—C7	120.58 (17)	C11—C12—H12B	109.8
C8—C7—C15	108.83 (16)	H12A—C12—H12B	108.3
C8—C7—C6	110.16 (16)	C13—C12—H12C	108.6
C15—C7—C6	112.73 (16)	C11'—C12—H12C	109.1
C8—C7—H7	108.3	C11—C12—H12C	138.6
C15—C7—H7	108.3	H12A—C12—H12C	71.7
C6—C7—H7	108.3	C13—C12—H12D	108.6
C13—C8—C9	118.77 (19)	C11'—C12—H12D	108.6
C13—C8—C7	123.07 (18)	C11—C12—H12D	75.4
C9—C8—C7	118.15 (18)	H12B—C12—H12D	136.4
O2—C9—C8	120.0 (2)	H12C—C12—H12D	107.5
O2—C9—C10	122.1 (2)	C8—C13—O1	122.63 (18)
C8—C9—C10	117.8 (2)	C8—C13—C12	126.9 (2)
C11—C10—C9	114.4 (2)	O1—C13—C12	110.45 (18)
C11'—C10—C9	119.7 (4)	N2—C14—O1	111.88 (18)
C11—C10—H10A	108.7	N2—C14—C15	127.66 (19)
C11'—C10—H10A	129.7	O1—C14—C15	120.45 (18)
C9—C10—H10A	108.7	C14—C15—N1	120.21 (18)
C11—C10—H10B	108.7	C14—C15—C7	123.32 (18)
C11'—C10—H10B	70.3	N1—C15—C7	116.47 (17)
C9—C10—H10B	108.7	N2—C16—H16A	109.5
H10A—C10—H10B	107.6	N2—C16—H16B	109.5
C11—C10—H10C	72.8	H16A—C16—H16B	109.5
C11'—C10—H10C	107.4	N2—C16—H16C	109.5
C9—C10—H10C	107.4	H16A—C16—H16C	109.5
H10B—C10—H10C	138.8	H16B—C16—H16C	109.5
C11—C10—H10D	136.3	O4—N1—O3	120.52 (18)
C11'—C10—H10D	107.4	O4—N1—C15	118.43 (18)
C9—C10—H10D	107.4	O3—N1—C15	121.05 (18)
H10A—C10—H10D	67.7	C14—N2—C16	125.1 (2)
H10C—C10—H10D	106.9	C14—N2—H2A	110.2 (17)
C10—C11—C12	112.5 (2)	C16—N2—H2A	124.7 (17)
C10—C11—H11A	109.1	C14—O1—C13	119.69 (16)
			
C6—C1—C2—C3	0.9 (3)	C10—C11'—C12—C13	−33.1 (11)
C1—C2—C3—C4	0.1 (3)	C10—C11'—C12—C11	57.6 (6)
C1—C2—C3—Cl1	−178.49 (17)	C10—C11—C12—C13	46.9 (3)
C2—C3—C4—C5	−0.9 (3)	C10—C11—C12—C11'	−58.2 (6)
Cl1—C3—C4—C5	177.67 (16)	C9—C8—C13—O1	175.11 (18)
C3—C4—C5—C6	0.7 (3)	C7—C8—C13—O1	−4.1 (3)
C2—C1—C6—C5	−1.1 (3)	C9—C8—C13—C12	−4.8 (3)
C2—C1—C6—C7	177.64 (19)	C7—C8—C13—C12	176.0 (2)
C4—C5—C6—C1	0.3 (3)	C11'—C12—C13—C8	20.5 (7)
C4—C5—C6—C7	−178.48 (18)	C11—C12—C13—C8	−20.3 (3)
C1—C6—C7—C8	−119.8 (2)	C11'—C12—C13—O1	−159.3 (6)
C5—C6—C7—C8	58.9 (2)	C11—C12—C13—O1	159.8 (2)
C1—C6—C7—C15	118.4 (2)	N2—C14—C15—N1	0.5 (3)
C5—C6—C7—C15	−62.8 (2)	O1—C14—C15—N1	−179.05 (18)
C15—C7—C8—C13	13.3 (3)	N2—C14—C15—C7	−179.6 (2)
C6—C7—C8—C13	−110.8 (2)	O1—C14—C15—C7	0.9 (3)
C15—C7—C8—C9	−166.00 (17)	C8—C7—C15—C14	−11.7 (3)
C6—C7—C8—C9	69.9 (2)	C6—C7—C15—C14	110.8 (2)
C13—C8—C9—O2	−174.9 (2)	C8—C7—C15—N1	168.27 (17)
C7—C8—C9—O2	4.4 (3)	C6—C7—C15—N1	−69.2 (2)
C13—C8—C9—C10	3.3 (3)	C14—C15—N1—O4	176.39 (19)
C7—C8—C9—C10	−177.4 (2)	C7—C15—N1—O4	−3.6 (3)
O2—C9—C10—C11	−156.9 (3)	C14—C15—N1—O3	−3.9 (3)
C8—C9—C10—C11	24.9 (3)	C7—C15—N1—O3	176.11 (18)
O2—C9—C10—C11'	159.0 (6)	O1—C14—N2—C16	4.2 (3)
C8—C9—C10—C11'	−19.1 (7)	C15—C14—N2—C16	−175.4 (2)
C11'—C10—C11—C12	57.4 (6)	N2—C14—O1—C13	−169.49 (18)
C9—C10—C11—C12	−50.4 (4)	C15—C14—O1—C13	10.1 (3)
C11—C10—C11'—C12	−59.2 (7)	C8—C13—O1—C14	−8.7 (3)
C9—C10—C11'—C12	33.9 (11)	C12—C13—O1—C14	171.21 (19)

### Hydrogen-bond geometry (Å, º)

Cg1 is the centroid of the pyran ring C7/C8/C13/O1/C14/C15.

**Table:**

*D*—H···*A*	*D*—H	H···*A*	*D*···*A*	*D*—H···*A*
N2—H2*A*···O3	0.90 (2)	1.86 (2)	2.599 (2)	137 (2)
C2—H2···O4^i^	0.93	2.53	3.420 (3)	160
C10—H10*A*···*Cg*1^ii^	0.97	2.75	3.515 (2)	136
C16—H16*B*···*Cg*1^iii^	0.96	2.76	3.577 (3)	144

Symmetry codes: (i) −*x*+3/2, *y*+1/2, −*z*+1/2; (ii) −*x*+2, −*y*+2, −*z*; (iii) −*x*+1, −*y*+1, −*z*.
